# Type 2 Diabetes Mellitus and Liver Disease: Across the Gut–Liver Axis from Fibrosis to Cancer

**DOI:** 10.3390/nu15112521

**Published:** 2023-05-29

**Authors:** Vittoria Manilla, Francesco Santopaolo, Antonio Gasbarrini, Francesca Romana Ponziani

**Affiliations:** 1 Digestive Disease Center—CEMAD, Fondazione Policlinico Universitario Agostino Gemelli IRCCS, 00168 Rome, Italy; 2 Translational Medicine and Surgery Department, Università Cattolica del Sacro Cuore, 00168 Rome, Italy

**Keywords:** diabetes, liver fibrosis, hepatocellular carcinoma (HCC), cholangiocarcinoma, insulin resistance (IR), non-alcoholic fatty liver disease (NAFLD), gut–liver axis, lypopolisaccharides (LPS), mitochondria, liver sinusoid endothelial cell (LSEC)

## Abstract

Type 2 diabetes mellitus is a widespread disease worldwide, and is one of the cornerstones of metabolic syndrome. The existence of a strong relationship between diabetes and the progression of liver fibrosis has been demonstrated by several studies, using invasive and noninvasive techniques. Patients with type 2 diabetes mellitus (T2DM) and nonalcoholic fatty liver disease (NAFLD) show faster progression of fibrosis than patients without diabetes. Many confounding factors make it difficult to determine the exact mechanisms involved. What we know so far is that both liver fibrosis and T2DM are expressions of metabolic dysfunction, and we recognize similar risk factors. Interestingly, both are promoted by metabolic endotoxemia, a low-grade inflammatory condition caused by increased endotoxin levels and linked to intestinal dysbiosis and increased intestinal permeability. There is broad evidence on the role of the gut microbiota in the progression of liver disease, through both metabolic and inflammatory mechanisms. Therefore, dysbiosis that is associated with diabetes can act as a modifier of the natural evolution of NAFLD. In addition to diet, hypoglycemic drugs play an important role in this scenario, and their benefit is also the result of effects exerted in the gut. Here, we provide an overview of the mechanisms that explain why diabetic patients show a more rapid progression of liver disease up to hepatocellular carcinoma (HCC), focusing especially on those involving the gut–liver axis.

## 1. Introduction

Type 2 diabetes mellitus (T2DM) is a proven independent risk factor for liver disease progression up to HCC development in patients affected by chronic liver disease of different etiologies [1]. However, the relationship between liver disease and diabetes is much more intricate than a simple linear relationship, and the two conditions appear to be closely interconnected. Given the pivotal role of the liver in glucose homeostasis regulation, it is not surprising that hepatic dysfunction leads to the development of insulin resistance (IR), and, in many cases, to a specific condition called hepatogenous diabetes [2]. Indeed, up to 80% of cirrhotic patients are affected by glucose metabolism disorder, regardless of the etiology [3]. Chronic hepatitis C virus (HCV) infection is associated with steatosis and IR in about half of patients; metabolic and cytopathic effects lead to the development of liver steatosis with the accumulation of intracellular fat, reducing glucose entrance into hepatocytes, inducing IR [4,5]. Additionally, tumor necrosis factor alpha (TNF-α) elevated plasma levels downregulate insulin-receptor substrate 1 (IRS-1) signaling, diminishing the translocation of glucose transporters to the plasma membrane; this reduces glucose uptake and increases blood glucose levels and insulin secretion [4]. Higher plasma levels of insulin on their hand entail the development of a certain grade of inflammation, modulated by the adipose tissue and the gut microbiota [5], being also capable to alter fatty acids pathway in the liver: insulin increases lipolysis and promotes de novo lipogenesis, with subsequent elevation of hepatic free fatty acid (FFA) influx, favoring fat accumulation and steatosis progression [6]. Persistent hyperglycemia leads also to a major production of advanced glycation end-products (AGEs), mainly derived from diet, which are able to increase oxidative stress and initiate hepatocyte damage and liver fibrosis [7,8].

Type 1 diabetes mellitus (T1DM), while always characterized by persistent hyperglycemia and systemic inflammation [9], does not share with T2DM the main pathogenic element, IR, which certainly plays a key role in triggering liver disease. For this reason, the literature on the role of T1DM in the development of liver disease, fibrosis, and cancer is not included in this review.

## 2. Epidemiology

### 2.1. Diabetes and Insulin Resistance in Non-Alcoholic Fatty Liver Disease (NAFLD)

Non-alcoholic fatty liver disease (NAFLD) has jumped in a few decades from being an unknown disease to becoming the most common chronic liver disease in the United States and is approaching to be a leading cause of liver transplantation [10]. Analyzing the global prevalence of NAFLD diagnosed through imaging methods (ultrasound, computed tomography scan, and magnetic resonance imaging/spectroscopy), it is strictly associated with obesity and metabolic syndrome, with Western lifestyle as main risk factor; furthermore, a genetic predisposition has been recognized in specific ethnic groups, such as Hispanics [11,12]. According to latest studies, the patatin-like phospholipase domain-containing protein 3 (PNPLA3) gene accounts for the largest fraction of genetic predisposition to NAFLD; in particular, the carriage of the I148M variant is associated with hepatic fat accumulation, a reduction in adiponectin, and a direct impact on adiposity and insulin resistance. The PNPLA3 I148M variant increases susceptibility to the whole spectrum of liver damage with progression to fibrosis up to hepatocellular carcinoma (HCC), and it is commonly considered a liver disease modifier [13]. Globally, the highest prevalence of NAFLD is reported in South America (32%), with country variation depending on the prevalence of obesity, and the Middle East; the lowest rate is reported in Africa. In Europe, approximately 25% of the population results to be affected, with a prevalence mirroring that of obesity. However, hepatic steatosis is not always a companion to obesity: “lean NAFLD” [14] can be surprisingly found in 10–20% of non-obese patients coming from Asia and America [15], who are predominantly men without classical risk factors, but can be affected by nonalcoholic steatohepatitis (NASH) and fibrosis in 61% and 55% of cases, respectively. Lean NAFLD prevalence accounts for 39% of cases of cryptogenic liver disease, and this number is probably underestimated [16]. Different studies tried to unearth risk factors in this specific population; those who are lean and affected by NAFLD are often younger, and, even if they have a lower prevalence of diabetes and metabolic syndrome than NAFLD overweight patients [17], they are insulin-resistant and have higher plasma triglyceride levels when compared with matched healthy controls [18]. Interestingly, lean NAFLD is very common in the Asian rural area, and, after adjustment for severity of visceral obesity in this population, rates of advanced liver fibrosis are similar between lean and obese NAFLD patients, even if with a concomitant lower metabolic burden in the first group [11]. Visceral adiposity measured by body mass index (BMI) and waist circumference can influence the manifestations of the metabolic syndrome but not the severity of liver damage in patients with NAFLD; altered glucose metabolism, instead, is predictive of liver fibrosis [19]. Lean NAFLD patients have a high visceral adiposity even when normotensive, normolipidaemic, and non-diabetic, and their adipose tissue is an important site involved in IR development [20]. Although NAFLD among patients with T2DM has a prevalence two to three times higher than in the general population [12], in a cohort of diabetic individuals with elevated liver tests, increased liver stiffness, or high liver fat percentage, NASH was found in 61% of biopsies, with a rate of advanced fibrosis measured by magnetic resonance elastography (MRE) of 14% [1]; nevertheless, even in patients with normal ALT levels, a high rate of advanced fibrosis could be found when glucose metabolism disorders were associated [21]. Not surprisingly, a similar rate of fibrosis was obtained with liver stiffness measurement (LSM) by vibration-controlled transient elastography [22]. Taken together, these findings showed the high burden of underdiagnosed liver disease in diabetic patients and opened the way to new urgent questions that need a response, the most relevant being whether NAFLD is the cause or consequence of diabetes [23], or what factors favored by diabetes might be driving the progression of liver disease.

### 2.2. Prevalence of HCC in NAFLD

The rapid progression towards advanced liver fibrosis and HCC development is the most worrisome event in patients diagnosed with NAFLD and diabetes. Measuring the hazard of HCC in cirrhotic patients, Yong et al. found it to be two times higher in the diabetic population (excluding those with chronic HCV infection), with the highest increase in HCC risk when diabetes was associated with NASH cirrhosis [24,25]. These findings lead the scientific community to recognize diabetes as an independent risk factor for developing HCC in patients affected by NASH cirrhosis [26]. As NAFLD is among the most common causes of chronic liver disease in Western countries [10], and the number of people with diabetes has more than tripled in a few decades [27], an evident consequence will be the progressive rise in the incidence of HCC, despite the decline of viral-related cases, especially in regions where the prevalence of obesity and diabetes is high [28]. 

Recently, innovative invasive and non-invasive methods for detecting steatosis and fibrosis have given a precious aid in understanding the burden of NAFLD in diabetic patients, allowing us to estimate its frequency and monitor the progression toward advanced fibrosis, cirrhosis, and HCC. Using vibration-controlled transient elastography, Lo Monaco et al. found that 15% of diabetic subjects screened and unknown to have NAFLD had moderate to advanced fibrosis (F2 or higher), suggesting again that even mild fibrosis (F1) in the setting of obesity and T2DM increases the risk of rapid liver disease progression [29]. Other studies based on liver biopsy also confirmed that patients affected by T2DM had higher rates of NASH and advanced fibrosis [30]. However, even risk assessment by grading liver fibrosis could be insufficient; in fact, in the NAFLD population, HCC could be found in absence of advanced fibrosis [31,32] and, in this case, diabetes represents the strongest independent risk factor [25,26].

## 3. How Insulin Resistance and Diabetes Favor Liver Fibrosis Progression

### 3.1. Insulin Resistance Is Involved in Hepatocyte Damage in NAFLD and NASH

As described above, a clear epidemiological link exists between diabetes and NAFLD in all its forms, and the role of IR in promoting liver fat accumulation is widely recognized. An increased prevalence of peripheral and adipose IR has been found not only in obese patients affected by NAFLD [33] but also in the lean NAFLD population without diabetes or metabolic syndrome. In the latter, plasma C-peptide levels are significantly higher across the insulin dose–response curve when compared to controls, and an increased visceral/subcutaneous abdominal fat ratio, as determined by magnetic resonance imaging (MRI), has been described [20]. Histological examination revealed how liver steatosis grade was inversely related to hepatic and skeletal muscle IR [34] and disclosed that mitochondrial involvement was associated with NASH, which was consistent with mitochondria pivotal function in lipid and glucose metabolism [35]. Indeed, in NASH, hepatocellular mitochondria showed paracrystalline inclusion bodies, were swollen, rounded, and often multilamellar with loss of cristae [36]. This also matches with the latest discoveries about genetic polymorphisms that drive NAFLD progression: the presence of the PNPLA3 I148M polymorphism, the rs641738 variant in the Membrane-bound O-acyltransferase domain containing 7-transmembrane channel-like 4 (MBOAT7-TMC4) locus, and the E167K Transmembrane 6 Superfamily Member 2 (TM6SF2) variant were correlated with increased oxidative stress of the endoplasmic reticulum, with subsequent alterations of mitochondrial ultrastructure and functions; this was clinically associated not only with IR but also with NAFLD progression toward severe forms of liver disease and HCC [37,38]. IR can be a stress factor that initiates hepatocyte damage, especially in carriers of the PNPLA3 I148M polymorphism [39], giving origin to a vicious circle. PNPLA3 I148M polymorphism is related to decreased levels of adiponectin [13], which drives adipose tissue–liver crosstalk; it promotes the production of anti-inflammatory cytokines such as interleukin-10 (Il-10) and regulates intracellular fat storage and metabolism, protecting liver from inflammation, fibrosis progression, and tumorigenesis [40]. On the contrary, the abnormal lipid peroxidation leads to lipotoxicity that damages the hepatocytes and promotes hepatic stellate cells (HSCs) proliferation; HSCs exert their pro-fibrogenic functions and are also associated with the release of pro-inflammatory cytokines and reactive oxygen species (ROS) that trigger Toll-like receptor-4 (TLR4) synthesis and the activation of liver macrophages [41]. Similar to TLR4, Toll-like receptor 2 (TLR2) also triggers inflammation while promoting pancreatic beta-cells dysfunction and diabetes development [42]. TLR2 is mainly activated by palmitate, one of the most abundant free fatty acids, and, after its inhibition, Zhang et al. demonstrated a decreased lipotoxicity in hepatic cells [43,44,45]. Peripheral number of macrophages and functions change, switching from an anti-inflammatory (M2) phenotype to a pro-inflammatory (M1) phenotype that produces, among other mediators, TNF-α and interleukin-6 (IL-6), eventually activating the nuclear factor-kappa B (NF-κB) pathway [41]. The latter balances proliferative and apoptotic processes, favoring liver damage progression, fibrosis, and tumorigenesis when not properly regulated [46]. It also leads to the activation of NLRP3 inflammasome with a consequent increased production of interleukin-1 β (IL-1β). On its hand, IL-1β further promotes insulin resistance, triggering TNF-α production and decreasing the phosphorylation of IRS-1, also contributing to β-cell failure and diabetes progression [47]. Not surprisingly, NLRP3 inflammasome has been linked to liver inflammation and fibrosis in mice. In liver disease of a different etiology, NLRP3 activation in hepatocytes and non-parenchymal cells results in pyroptotic cell death and HSCs activation, responsible for collagen deposition and fibrosis [48].

### 3.2. Insulin Resistance Promotes Endothelial Dysfunction Contributing to Liver Fibrosis Progression

IR promotes the alteration of hepatic fat storage and hepatocytes inflammatory injury, but how is it involved in the systemic alterations that lead to liver disease progression up to carcinogenesis? Of course, diabetes causes systemic microvascular and macrovascular alterations, but liver sinusoidal microvascular damage has always been considered rare [49,50]. Liver sinusoidal endothelial cells (LSECs) exhibit unique phenotypic characteristics that include open fenestrae and lack of a basement membrane. Given the critical role of the interface between hepatocytes and blood flow, these features allow LSECs to communicate with different cell types and molecules to preserve the hepatic microenvironment [51,52]. The importance of their continuous interplay with immune cells has been demonstrated in acute and chronic liver injury of different etiology [51], with different harmful stimulations leading LSECs to lose fenestrae and develop a basement membrane in a process called “capillarization”, with subsequent increase in intrahepatic vascular resistance [53]. Insulin interacts with LSECs in different ways: physiologically, it promotes nitric oxide (NO) production through the activation of the phosphatidylinositol 3-kinase (PI3K)/AKT/endothelial NO synthase (eNOS) signaling pathway, making LSECs the main source of endothelium-derived NO, which is an important modulator of vascular tone [54]. However, as seen in mice fed with a high fat diet (HFD), when IR develops, the beneficial vascular effects of insulin are impaired, with the progressive development of endothelial dysfunction. Interestingly, IR can be found in LSECs before the development of other signs of NAFLD, manifesting with decreased eNOS activity and an upregulation of inducible NOS (iNOS), which is responsible for the increase in intrahepatic vascular resistance [55]. As reported above, persistent hyperglycemic state also leads to the initiation and progression of non-enzymatic glycation, which generates a heterogeneous group of molecules known as AGEs. AGEs bind their cellular receptor, RAGE, which activates multiple signaling pathways that enhance oxidative stress and inflammation [56]. Interestingly, LSECs and Kuppfer cells are the major cellular sites of AGEs uptake and clearance, and liver microcirculatory dysfunction impairs AGEs’ metabolism, leading to further increases in their plasma concentrations in patients with liver diseases [57]. These mechanisms could explain the early development of portal hypertension observed in NAFLD rats and humans even before significant fibrosis [58,59]. The early capillarization of LSECs may also speed-up the onset of unfavorable features typical of a pro-oncogenic microenvironment, through the activation of pathways linked to angiogenesis, coagulation, and fibrinolysis; this can lead to an altered marker expression profile on LSECs, which can potentially be used as therapeutic target in HCC [51]. Furthermore, the increased visceral adiposity that accompanies IR [19,20] alters the production of the insulin growth factor-1 (IGF-1) and insulin growth factor-2 (IGF-2), whose levels have been linked to neoangiogenesis and the development of different types of malignancies, including liver cancer [25,60,61]. Levels of inflammatory cytokines such as IL-6, TNF-α, and iNOS increase too, fueling systemic low-grade inflammation, a typical hallmark of various metabolic disorders [62]. Low-grade inflammation, though clinically silent, sustains endothelial dysfunction [63] and alters the immune system response, increasing the oncogenic risk [64].

## 4. Interplay between Insulin Resistance and the Gut–Liver Axis

### 4.1. From Insulin Resistance to Metabolic Endotoxemia

All the mechanisms described above help the progression of liver disease and recognize IR as a driver. However, where this insidious process begins is still poorly understood. Considering the key role of dietary habits in diabetes onset [65], it is reasonable to speculate that the intestinal barrier could be a major protagonist. The gut microbiota is part of the external layer of the barrier coating the intestinal lumen [66] and lies on a mucus layer rich in defensive proteins, which keeps microorganisms distant from the intestinal epithelium. In physiologic conditions, intestinal bacteria promote mucus secretion by goblet cells and influence mucus stratification through the production of short-chain fatty acids (SCFAs), derived from dietary fibers [67,68]. Beneath mucus, the intestinal epithelium is directly in contact with the gut vascular barrier (GVB), which consists of a single layer of endothelial cells connected through tight junctions and adherens junctions that tightly control the paracellular flow of solutes and fluids in cooperation with pericytes and fibroblasts [69]. The intestinal barrier, including the GVB, is the gatekeeper of the interface between the host and the external environment, as also confirmed by the well-recognized relationship between its dysfunction and multiple diseases [67]; the gastrointestinal tract also contains up to 70% of the entire lymphocyte population, making it the largest immunological organ in the body. Specifically, within the intestinal barrier, the lamina propria contains dendritic cells, which are important antigen presenting cells and gut-associated lymphoid tissue (GALT), which includes Peyer’s patches, lamina propria-lymphocytes, and intraepithelial lymphocytes. Starting with the immune cells found in its layers, the characteristics of the intestinal barrier are dynamically modulated by microbial antigens and metabolites, which, therefore, shape the systemic innate and adaptive immune response [70]. Endotoxin (lypopolisaccharides—LPS) translocation in the systemic circulation due to gut dysbiosis cis one of the main drivers of immunomodulation: once entered into the bloodstream, LPS binds to the CD14/TLR4 complex on the macrophage’s surface and favors M1 macrophage proliferation with subsequent production of TNFα, IL-1β, and IL-6, which contribute to insulin resistance by phosphorylating serine of IRS-1; as a result, insulin signaling is reduced, triggering insulin resistance [71]. The increased circulation of proinflammatory molecules leads to what we define as metabolic endotoxemia, which fuels low-grade inflammation [72]. Not surprisingly, diabetic patients show higher LPS serum levels compared with non-diabetic lean or obese subjects, and they are even higher in carriers of diabetes-related complications [73]. Gastro-intestinal disorders as gastroparesis and/or small intestine bacterial overgrowth (SIBO) are common in long standing diabetes [74], and, in this context, the metabolic endotoxemia increase is justified by two fundamental mechanisms: the impaired intestinal permeability driven by gut dysbiosis [73] and the reduced clearance of LPS by liver immune cells, whose function is impaired by hyperinsulinemia [75]. Martin et al. recently showed how hyperglycemia by itself can disrupt the intestinal barrier through the increased transport of glucose into mice intestinal cells, mediated by the GLUT-2 receptor, leading to an enteric impaired response to insulin; according to their study, higher glycated hemoglobin (HbA1c) serum levels are associated with increased levels of TLR4 ligands [76], an indirect marker of circulating LPS [77]. These results are not in contrast with the fact that patients with T2DM show a low enteric intracellular insulin response with a concomitant decrease in acetylcholine phosphorylation and an increase in chylomicron production, both driving intestinal lipotoxicity and oxidative stress [78], leading to inflammation and increased risk of liver fibrosis progression and hepatocarcinogenesis [79].

### 4.2. Dysbiosis and Metabolic Endotoxemia as a Trigger of Insulin Resistance and Diabetes: A Vicious Circle

Metabolic endotoxemia is not only a late complication of T2DM but, rather, is considered a trigger of IR. Cani et al. demonstrated that acutely feeding mice with HFD increased LPS, promoting the development of liver IR. On the other side of the intestinal barrier, HFD administration was associated with a reduction in *Bacteroides*, *E. rectale*, *C. coccoides*, and *Bifidobacteria*, suggesting that the gut microbiota also has a role in regulating insulin sensitivity [80]. This hypothesis was supported by an experimental study from Bäckhed et al., highlighting that gut microbiota transplantation from conventionally fed mice to adult germ-free animals produced a 60% increase in body fat content and IR, despite low food intake [81]. Multiple mechanisms, including GVB preservation, allowed the gut microbiota to regulate lipid and glucose homeostasis. Food, and, in particular, dietary AGEs, the abundance of which depends on food processing and preparation, could affect the production of gut-microbiota-derived metabolites, such as SCFAs [82]. Indeed, acetate, propionate, and butyrate derive from the fermentation of polysaccharides, and their quality and quantity are influenced by the amount of dietary non-digestible carbohydrates and by the gut microbial composition [83]. Among them, butyrate plays an important role in preserving intestinal integrity through the regulation of tight junctions proteins expression and mucus production, also exerting anti-inflammatory properties by increasing the number of regulatory T cells (Tregs) and reducing adipose tissue macrophage infiltration, thus improving insulin sensitivity. Not surprisingly, butyrate production is associated with an improved insulin response and GVB integrity [84,85]. Diabetic patients show a decreased abundance of butyrate-producing bacteria from the *Ruminococcaceae* family [86,87,88], *Roseburia* and *Faecalibacterium prausnitzii*; these features have been used to identify patients with T2DM in a cohort of European women, together with the decrease in *Lactobacillus gasseri*, which has a well-known role in regulating lipid and glucose metabolism [89,90,91]. In regard to the other SCFAs, acetate increases fatty acid oxidation and energy expenditure, whereas propionate represents a fundamental substrate for gluconeogenesis [92]; SCFAs also increase circulating levels of glucagon-like peptide-1 (GLP-1) and peptide YY (PYY) while decreasing TNF-α, with the consequent modulation of satiety and reduction in systemic inflammation [93]. Eventually, an impaired production of propionate appeared to be related to an increased risk of T2DM [84,85]. Another group of metabolites produced by the gut microbiota closely linked to IR are leucine, isoleucine, and valine, branched chain amino acids (BCAAs). Animal studies demonstrated that BCAA supplementation during HFD reduced food intake and body weight but did not prevent the development of IR. In humans, BCAAs emerged as predictors of future diabetes development, and it has been hypothesized that *Prevotella copri* and *Bacteroides vulgatus* could be involved in this process by increasing BCAAs biosynthesis, promoting IR [94]. BCAAs act by activating the mammalian target of rapamycin (mTOR) within the phosphoinositide-3-kinase-protein kinase B (PI3K-Akt) signaling pathway [95], which is the route used by insulin to regulate glucose metabolism, cell growth, and proliferation [96,97]. Aromatic amino acid (tryptophan, phenylalanine, and tyrosine) derivatives produced by gut microbiota play an ambivalent role in lipid and glucose metabolism, limit the intestinal translocation of harmful bacterial products, such as LPS, and exert most of their effects on hepatic metabolism by exploiting the aryl hydrocarbon receptor (AhR). AhR is activated by bacterial metabolites derived from tryptophan such as indole-3-acetic acid, resulting in the reduction of lipid intracellular accumulation and the modulation of hepatic inflammation. In addition, indole regulates GLP-1 secretion from intestinal L-cells [83,98], which play a key role in glucose–insulin interplay. Indeed, GLP-1 receptor agonists, widely used in diabetes treatment, showed encouraging results in improving liver fat accumulation, inflammation, and fibrosis [99,100,101], also appearing as effective in decreasing HCC tumor cells migration [102,103]. On the contrary, phenylacetic acid—derived from phenylalanine—is positively associated with steatosis severity in obese women, and its administration to human liver cells induces lipid accumulation and alters the expression of genes involved in lipid and glucose metabolism. The gut microbiota also produce secondary biliary acids (BA) [83] that control inflammation, glucose, and lipid homeostasis via the nuclear farnesoid X receptor (FXR) and the Takeda G protein coupled receptor 5 (TGPR5). The liver synthesizes and conjugates primary BA that are secreted in the intestinal lumen, reaching the large intestine. Here, they are deconjugated and transformed into secondary BA by intestinal bacteria, promoting FXR activation. This process also inhibits lipogenesis, thus decreasing intracellular fat accumulation, hepatic inflammation, and fibrosis. For these reasons, FXR agonists such as obeticholic acid can be a promising therapeutic tool in the NASH population, although mixed results on insulin sensitivity have been obtained in clinical trials, and data on the increased risk of HCC need further cautious evaluation [104,105].

## 5. Role of Insulin Resistance in the Development of Primary Liver Tumors

### 5.1. Hepatocellular Carcinoma

As reported above, T2DM is recognized as an independent risk factor for HCC development [26], even in the absence of advanced fibrosis [31,32]. IR is directly related to liver fat accumulation and the mitochondrial impairment driven by lipotoxicity [33,34], thus increasing oxidative stress [36,37]. Two proteins involved in the mitochondrial fusion process, mitofusin-1 (MFN-1) and mitofusin-2 (MFN-2), seem to play an opposite role in maintaining insulin sensitivity. In particular, mice on HFD showed reduced hepatic MFN2 in association with IR and oxidative stress development, and the loss of heterozygosity in the MFN2 gene has been demonstrated in HCC, with low MFN2 expression being correlated with worse survival. On the contrary, MFN-1 deficiency seems to exert a protective role against diabetes, but its expression is reduced in HCC tissue compared to adjacent non neoplastic tissue, being inversely related with epithelial-to-mesenchymal transition (EMT), vascular invasion, and poor prognosis [106]. At the same time, lipotoxicity-induced cellular damage triggers TLR4 synthesis and liver macrophages activation [41], increasing pro-inflammatory cytokines production and leading to an inappropriate activation of the NF-κB pathway, which plays a pivotal oncogenic role [46]. Indeed, low-grade inflammation, typical of the diabetic population, is sustained by intrahepatic lipotoxicity, adipose tissue dysfunction [62], and metabolic endotoxemia derived from intestinal barrier disruption, being probably the major factor responsible for HCC development in the absence of cirrhosis, which accounts for about 8% of HCC cases in NAFLD [18]. As further proof, endotoxin levels are increased in the portal and peripheral blood of patients with HCC, triggering TLR-4 activation, which promotes EMT and cancer progression [107]. EMT and neoangiogenesis are also promoted by IGF-impaired production that accompanies IR [108,108]. In rat models of early HCC, IGF-2 was expressed in the cytoplasm of both precancerous liver cells and malignant hepatocytes, but also in the rough endoplasmic reticulum and mitochondria of malignant hepatocytes, and seemed to promote hepatocytes proliferation by using both paracrine and autocrine mechanisms [61]. The IR effect on LSECs’ capillarization [55] decreased oxygen diffusion into the space of Disse and induced hepatocytes chronic ischemia, fueling inflammation and damaging DNA, with a subsequent and progressive loss of cellular differentiation [109] up to the appearance of cellular markers typical of neoplastic liver tissue [51]. Gut dysbiosis also takes part to the process of hepatocarcinogenesis related to diabetes by promoting intestinal barrier disruption and metabolic endotoxemia [110,111], and it is reasonable to speculate that multiple microorganisms exert a crucial role in this process, as proven by the protective effect against HCC demonstrated by certain gut microbiota modulators [112]. However, further studies are needed to better understand the relationship between gut microbiota, diabetes, and HCC.

### 5.2. Cholangiocarcinoma

T2DM is a strong risk factor for cholangiocarcinoma (CCC) development, especially for its intrahepatic form (iCCC), and is associated with a poorer prognosis [113,114]. Not surprisingly, iCCC incidence is rising in parallel with that of HCC [115], to the point that the repetitive dosage of gamma-glutamyl transferase and CA 19.9 has been suggested for patients’ screening [116]. Inflammation driven by IR is certainly one of the most important pathways involved in CCC onset, especially in those who show bile duct involvement in NAFLD/NASH disease [117]. In this setting, the cJun NH2-terminal kinase (JNK) stress-signaling pathway, involved in BA metabolism, has recently gathered attention not only due to the improvement in steatosis and IR obtained after its inhibition [118] but also because JNK deficiency leads to an increased risk of biliary cells inflammation and the development of intrahepatic cholangiocarcinoma, as demonstrated in rats [119]. Oral anti-diabetic medications may also play a crucial role in modifying the development and progression of CCC. In particular, growing evidence suggests the involvement of incretin-based therapy in CCC development, as suggested by the increase in glucagon-like peptide 1 (GLP-1) receptor expression during cholestasis and malignant transformation of bile duct epithelium, but also by its anti-apoptotic effect on normal cholangiocytes. On the contrary, metformin decreases the risk of CCC by acting on the mTOR/AMPK pathway and suppresses the nuclear translocation of the Signal transducer and activator of transcription 3 (STAT3) and the NF-kB pathway, suggesting a promising role in CCC chemoprevention and treatment [120].

## 6. Conclusions

**Figure 1:**
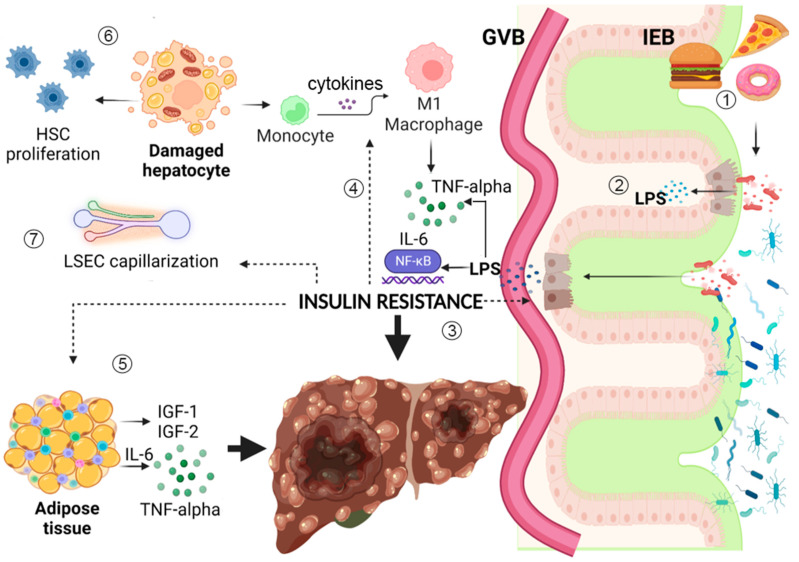
Insulin resistance (IR) is linked to multiple pathways that synergically lead to liver fibrosis and hepatocarcinogenesis. (1) HFD and dietary AGEs promote gut dysbiosis and IEB disruption. They alter the metabolic functions and immune modulating capacities of the gut microbiota, increasing LPS translocation into the bloodstream. (2) LPS binds to TLR-4/CD14 complex with the consequent activation of NF-kB, triggering metabolic endotoxemia and low-grade systemic inflammation that damages the liver and promotes carcinogenesis. (3) Inflammation and gut dysbiosis promote IR, which exerts direct damage on enterocytes, in association with hyperglycemia, increasing intracellular oxidative stress and epithelial intestinal permeability. (4) IR acts on the immune system, stimulating macrophage polarization to M1 phenotype, with subsequent production of multiple pro-inflammatory cytokines, such as TNF-alpha and IL-6, fueling low-grade inflammation. (5) Adipose tissue responds to IR modifying resident immune cells phenotype and takes part to the development of a pro-inflammatory microenvironment, also producing IGF-1 and IGF-2, which impair cell proliferation and angiogenesis, favoring hepatocarcinogenesis. (6) IR directly acts on hepatocytes altering intracellular fat storage, promoting lipotoxicity and oxidative stress with subsequent mitochondrial dysfunctional alterations; this worsens IR, damages hepatocytes, and increases HSC proliferation, favoring fibrosis development. (7) IR alters LSECs function, leading to sinusoid capillarization, thus increasing intrahepatic vascular resistance and leading to a further alteration of hepatic intercellular communication and microenvironment, increasing risk of uncontrolled cellular proliferation up to hepatocarcinogenesis. Figure created with Biorender.com. Dotted arrows represent insulin resistance direct action on different tissues and cell types.

The relationship between diabetes and liver disease progression is complex and paves the way to multiple questions that could help to find effective strategies to stop this insidious vicious circle, if answered. Even if it is not clear yet where the process begins, visceral adiposity certainly plays a key role in initiating IR and subsequent liver injury, even in the absence of increased BMI or an overt diagnosis of diabetes or metabolic syndrome, also conferring an increased risk of NAFLD, especially in PNPLA3 carriers [20,121]. IR, on its hand, drives early LSECs capillarization, contributing to the development of endothelial dysfunction and portal hypertension, even in the absence of fibrosis, worsening liver damage in the long term [58] and providing fertile ground for the development of HCC [44] (Figure 1). IR, endothelial dysfunction, and increased visceral adiposity fuel low-grade systemic inflammation together with metabolic endotoxemia, which appears as a consequence of gut dysbiosis and intestinal barrier dysfunction, probably driven by certain diet components such as fats or AGEs [82]. The contemporary activation of multiple pro-inflammatory pathways leads to permanent alterations that promote liver fibrosis and HCC. Considering primary liver tumors, the existence of a clear association between T2DM and CCC [113,114] deserves further studies to better understand the process of oncogenesis and improve not only preventive strategies but, more importantly, patients’ outcomes. Regarding the role of the gut microbiota in this intricate context, their fundamental immunomodulatory actions and established involvements in metabolic functions such as fat and glucose homeostasis regulation [70,71] open the way to a huge challenge for the future: the integration of data from both sides of the intestinal barrier. The goal will be to uncover the most important steps linking IR to liver disease progression, as well as to find new, effective interventions based also on compositional and functional modulation of the gut microbiota or restoration of gut barrier integrity. 

## Abbreviations

HFD	High-fatty diet
AGEs	advanced glycation end products
HSC	hepatic stellate cells
LSEC	liver sinusoid endothelial cell
TNF-alpha	tumor necrosis factor-alpha
Il-6	interleukin-6
LPS	lypopolisaccharides
nf-kB	nuclear factor-kappa-B
IGF-1	insulin growth factor-1
IGF-2	insulin growth factor-2
GVB	gut vascular barrier
IEB	intestinal epithelial barrier
